# Analysis of Cytomegalovirus Infection Affecting the Stomach

**DOI:** 10.1155/grp/4842972

**Published:** 2026-05-08

**Authors:** Yang Diao, Ye Zong, Rui Xu, Guangyong Chen, Xun Yang, Haiying Zhao

**Affiliations:** ^1^ Department of Gastroenterology, Beijing Friendship Hospital, Capital Medical University, Beijing, China, ccmu.edu.cn; ^2^ State Key Laboratory of Digestive Health, Beijing, China; ^3^ National Clinical Research Center for Digestive Disease, Beijing, China; ^4^ Beijing Key Laboratory of Early Gastrointestinal Cancer Medicine and Medical Devices, Beijing, China; ^5^ Department of Pathology, Beijing Friendship Hospital, Capital Medical University, Beijing, China, ccmu.edu.cn

**Keywords:** cytomegalovirus, gastritis, immunosuppression

## Abstract

**Background:**

The incidence of cytomegalovirus (CMV) infection is increasing. This study explored the clinical characteristics, diagnosis, treatments, and prognosis of gastric lesions caused by CMV infection.

**Methods:**

This is a systematic review of the literature on CMV infection involving the stomach.

**Results:**

A total of 39 English publications reporting 44 cases of CMV gastritis were identified through literature retrieval. Clinically, patients with CMV gastritis commonly presented with gastrointestinal symptoms, including abdominal pain, nausea and/or vomiting, and gastrointestinal bleeding. Endoscopic findings most frequently included mucosal ulcers (54.55%), erosions (27.27%), and erythema (20.45%). Most patients had a history of immunosuppression, such as AIDS, postorgan transplantation, cancer chemotherapy, or long‐term use of corticosteroids and immunosuppressants. The diagnosis was primarily confirmed by endoscopic biopsy combined with CMV DNA quantitative testing. The majority of patients improved after antiviral therapy, while a small subset experienced spontaneous resolution. However, a few cases (2.27%) progressed to gastric cancer, and some (4.55%) resulted in death.

**Conclusion:**

In patients with a history of immunosuppression presenting with gastrointestinal symptoms such as abdominal pain, nausea, vomiting, or gastrointestinal bleeding, CMV gastritis should be considered in the differential diagnosis. Endoscopic biopsy with histopathological examination plays a crucial role in confirming the diagnosis.

## 1. Introduction

Cytomegalovirus (CMV), a large (∼200 nm) DNA virus of the Herpesviridae family, is one of the biggest known animal viruses. Being an important opportunistic pathogen, primary CMV infection is typically asymptomatic; however, reactivation of latent CMV infection in immunocompromised individuals can be life‐threatening [1]. Patients with inflammatory bowel disease (IBD) are susceptible to intestinal CMV infection; however, gastric involvement by CMV remains uncommon even among immunocompromised individuals [2]. This study presents a systematic review of CMV gastritis literature. We summarize the clinical characteristics including manifestations, diagnostic approaches, therapeutic strategies, and outcomes, aiming to enhance clinical awareness and provide practical references for physicians and researchers.

## 2. Literature Search Strategy

A systematic literature search was conducted in PubMed using the keywords “cytomegalovirus” combined with “gastritis” or “gastric” for articles published between April 1980 and August 2025.

## 3. Results

From 560 retrieved articles, 39 studies (44 CMV gastritis cases) met the inclusion criteria.

### 3.1. Baseline Characteristics

Of the 44 patients, 25 (56.82%) were male and 19 (43.18%) were female, with ages ranging from 20 to 80 years (mean 52.07 years, Table 1). Immunosuppression was present in 37/44 (84.09%) CMV gastritis cases, comprising AIDS (15.91%), posttransplant (22.73%), and chemotherapy/immunosuppressant use (45.45%) (Table 2).

**Table 1:** Baseline demographic of patients with CMV gastritis.

	Cases (*n*)	Percentage (%)
Gender		
Male	25	56.82
Female	19	43.18
Age group (years)		
< 18	0	0
18–29	2	4.55
30–39	12	27.27
40–49	9	20.45
50–59	4	9.09
60–69	6	13.64
70–79	10	22.73
≥ 80	1	2.27

**Table 2:** Comorbidities in patients with CMV gastritis.

Comorbidities		Cases (*n*)	Management	Reference
HIV/AIDS		7	Antiretroviral therapy (ART)	[2–4]
Posttransplant	Renal transplantation	8	Immunosuppressive therapy	[5–11]
	Liver transplantation	1	Immunosuppressive therapy	[12]
	Bone marrow transplantation	1	Immunosuppressive therapy	[13]
Neoplasm	Non‐Hodgkin lymphoma	5	‐R‐CHOP chemotherapy and radiotherapy (*n* = 2)‐CHOP chemotherapy (*n* = 1)‐Immunochemotherapy with bendamustine and rituximab (*n* = 1)‐MACOP‐B chemotherapy (*n* = 1)	[14–18]
	Hodgkin lymphoma	1	Unspecified chemotherapy	[3]
	Malignant melanoma	3	‐ Surgery + pembrolizumab (*n* = 2)‐ Pembrolizumab (*n* = 1)	[19–21]
	Colorectal cancer	2	‐Ipilimumab + nivolumab (*n* = 1)‐Surgery + adjuvant chemotherapy (*n* = 1)	[22, 23]
	Clear cell renal cell carcinoma	1	Nivolumab	[24]
	Bladder cancer	1	Surgery + gemcitabine and cisplatin chemotherapy	[23]
	T cell leukemia	1	CHOP + methotrexate	[25]
Autoimmune disorders	Multiple sclerosis	1	Natalizumab	[26]
	Gastroduodenal sarcoidosis	1	Prednisone	[27]
	Pulmonary fibrosis	1	Prednisone	[28]
	Pemphigus	1	Prednisone	[29]
	Rheumatoid arthritis	1	NSAIDs and methotrexate	[30]
	*Helicobacter pylori*	2	*H. pylori* eradication (*n* = 2)	[21, 31]
Infection	Epstein–Barr virus	2	Not reported (*n* = 2)	[10, 32]
	SARS‐CoV‐2	1	Prednisone + tocilizumab + remdesivir	[1]
	Herpes simplex virus	1	Not reported	[10]
	*Strongyloides stercoralis*	1	Ivermectin	[6]
Unclassified	End‐stage renal disease	1	Hemodialysis	[33]

Abbreviations: CHOP, cyclophosphamide, doxorubicin, vincristine, prednisone; MACOP‐B, methotrexate, doxorubicin, cyclophosphamide, vincristine, prednisone, bleomycin; R‐CHOP, rituximab, cyclophosphamide, doxorubicin, vincristine, prednisone.

### 3.2. Clinical Manifestations

The predominant clinical presentation was abdominal pain (65.91%, 29/44), including three cases (6.82%) exhibiting characteristic positional epigastric pain described as improvement in supine position and exacerbation when sitting, standing, or walking [7]. Other common symptoms included nausea and/or vomiting (43.18%, 19/44), gastrointestinal bleeding (20.45%, 9/44), and decreased appetite (15.91%, 7/44). Less frequent manifestations comprised fever and weight loss (13.64%, 6/44), diarrhea (11.36%, 5/44), dysphagia (9.09%, 4/44), fatigue (6.82%, 3/44), abdominal distension (4.55%, 2/44), and constipation (2.27%, 1/44). Notably, gastric perforation occurred in 4.55% (2/44) of cases (Table 3).

**Table 3:** Clinical manifestations in patients with CMV gastritis.

Clinical manifestation	Cases (*n*)	Percentage (%)	Reference
Abdominal pain	29	65.91	[1, 3–9, 11, 13, 16, 17, 19–28, 30, 33–35]
Nausea and/or vomiting	19	43.18	[1–4, 6, 7, 17, 19–24, 26, 27, 32, 33, 36]
Gastrointestinal bleeding	9	20.45	[3–5, 12, 14, 15, 24, 28, 35]
Decreased appetite	7	15.91	[7, 10, 18, 31–33, 36]
Weight loss	6	13.64	[3, 5, 10, 18, 30, 36]
Fever	6	13.64	[4, 8, 11, 17, 32, 37]
Diarrhea	5	11.36	[3, 8, 11, 12, 16]
Dysphagia	4	9.09	[1, 3, 23]
Fatigue	3	6.82	[4, 30, 35]
Abdominal distension	2	4.55	[34, 38]
Gastric perforation	2	4.55	[4, 38]
Constipation	1	2.27	[38]

### 3.3. Endoscopic Findings

Endoscopy revealed gastric ulcers (54.55%) as the predominant finding, followed by erosions (27.27%) and erythema (20.45%). Nodular mucosal changes (9.09%, 4/44) were less frequently observed (Table 4).

**Table 4:** Endoscopic findings in patients with CMV gastritis.

Endoscopic findings	Cases (*n*)	Percentage (%)	Reference
Ulcer	24	54.55	[1, 3–5, 10, 12–17, 21, 23, 25, 28, 30, 31, 33, 36–38]
Erosion	12	27.27	[5, 8, 9, 13, 15, 24, 26, 32, 34, 36, 37, 39]
Erythema	9	20.45	[4, 5, 17, 22, 26, 28, 29, 31]
Nodule	4	9.09	[3, 11, 23, 27]

### 3.4. Diagnostic Methods

The diagnosis of CMV gastritis was confirmed in the majority of cases (95.45%, 42/44) by endoscopic biopsy. Histopathological examination revealed characteristic cytomegalic inclusions in 59.09% (26/44) of cases, while immunohistochemical (IHC) staining for CMV was positive in 45.45% (20/44). In the remaining 4.55% of patients (2/44), the diagnosis was established through quantitative CMV DNA PCR testing.

### 3.5. Treatments and Outcomes

The majority of patients (70.45%, 31/44) showed clinical improvement following medical therapy. Specifically, 59.09% (26/44) responded to ganciclovir and/or valganciclovir; 4.55% (2/44) improved with acid suppression therapy; 2.27% (1/44) benefited from CMV immunoglobulin; 2.27% (1/44) improved with symptomatic treatment alone; 2.27% (1/44) experienced spontaneous resolution. Notably, one patient (2.27%) progressed to gastric cancer. Despite receiving antiviral therapy, one patient (2.27%) ultimately died unfortunately. Another patient (2.27%) died following surgical intervention for gastric perforation complicated by acute peritonitis (Table 5).

**Table 5:** Management and outcomes of patients with CMV gastritis.

Management	Cases (*n*)	Outcomes	Reference(s)
Ganciclovir	20	‐Improved (*n* = 16)‐Not reported (*n* = 2)‐Progression to gastric cancer (*n* = 1)‐Death (*n* = 1)	[1–4, 7, 8, 11, 16, 18, 19, 21, 23, 24, 27, 29–31, 34, 35, 37]
Valganciclovir	3	Improved (*n* = 3)	[12, 26, 33]
Ganciclovir + valganciclovir	7	Improved (*n* = 7)	[5, 6, 9, 17, 20, 22]
Acid suppression	2	Improved (*n* = 2)	[13, 36]
CMV immunoglobulin	1	Improved	[25]
Supportive care (unspecified)	1	Improved	[32]
No treatment	1	Improved	[39]
Laparotomy	1	Death	[38]

## 4. Discussion

CMV gastritis represents an opportunistic infection that predominantly occurs in immunocompromised populations, including patients with AIDS, posttransplant status, cancer chemotherapy, or prolonged corticosteroid/immunosuppressant use. However, previous retrospective studies have demonstrated that approximately 17.9% of CMV gastritis patients exhibit immunocompetent status [40]. Our systematic review revealed that 11.36% of cases occurred in immunocompetent patients. Furthermore, the rising utilization of immunotherapeutic regimens has been correlated with an increasing incidence of CMV infections [19]. Therefore, clinicians should maintain a high index of suspicion for CMV coinfection. Although positional epigastric pain has been recognized as a characteristic clinical manifestation of CMV gastritis [8], the condition generally lacks pathognomonic symptoms. Patients may present with various nonspecific gastrointestinal symptoms, including epigastric pain, fever, nausea/vomiting, and gastrointestinal bleeding. Endoscopic features of CMV gastritis, as observed in our study and prior reports [29], typically include mucosal ulcers/erosions but can range from diffuse erythema and nodules to pseudotumors or normal mucosa.

**Figure 1:**
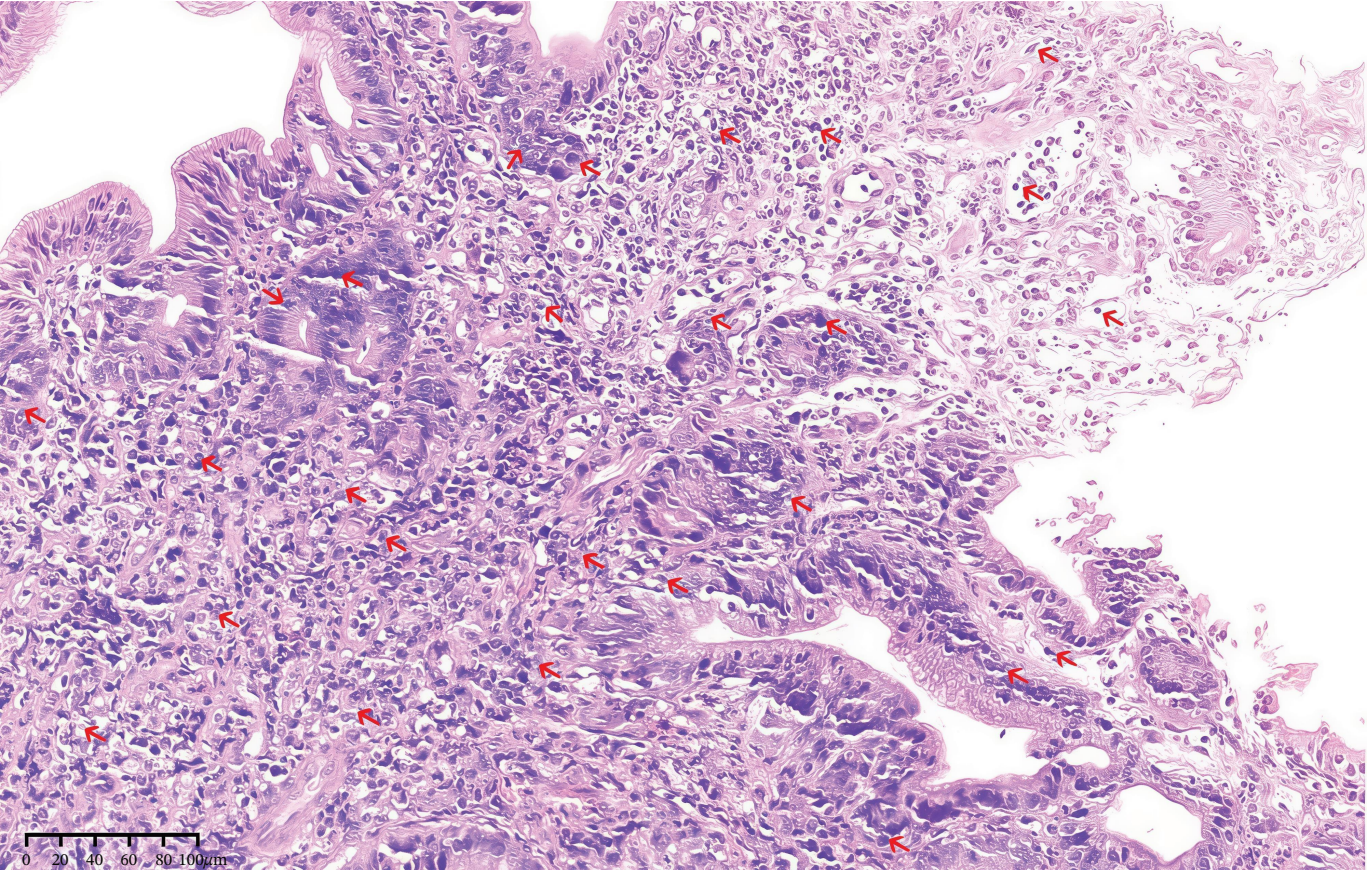
Foci of cells with enlarged nuclei in epithelium and stroma (HE ×200).

**Figure 2:**
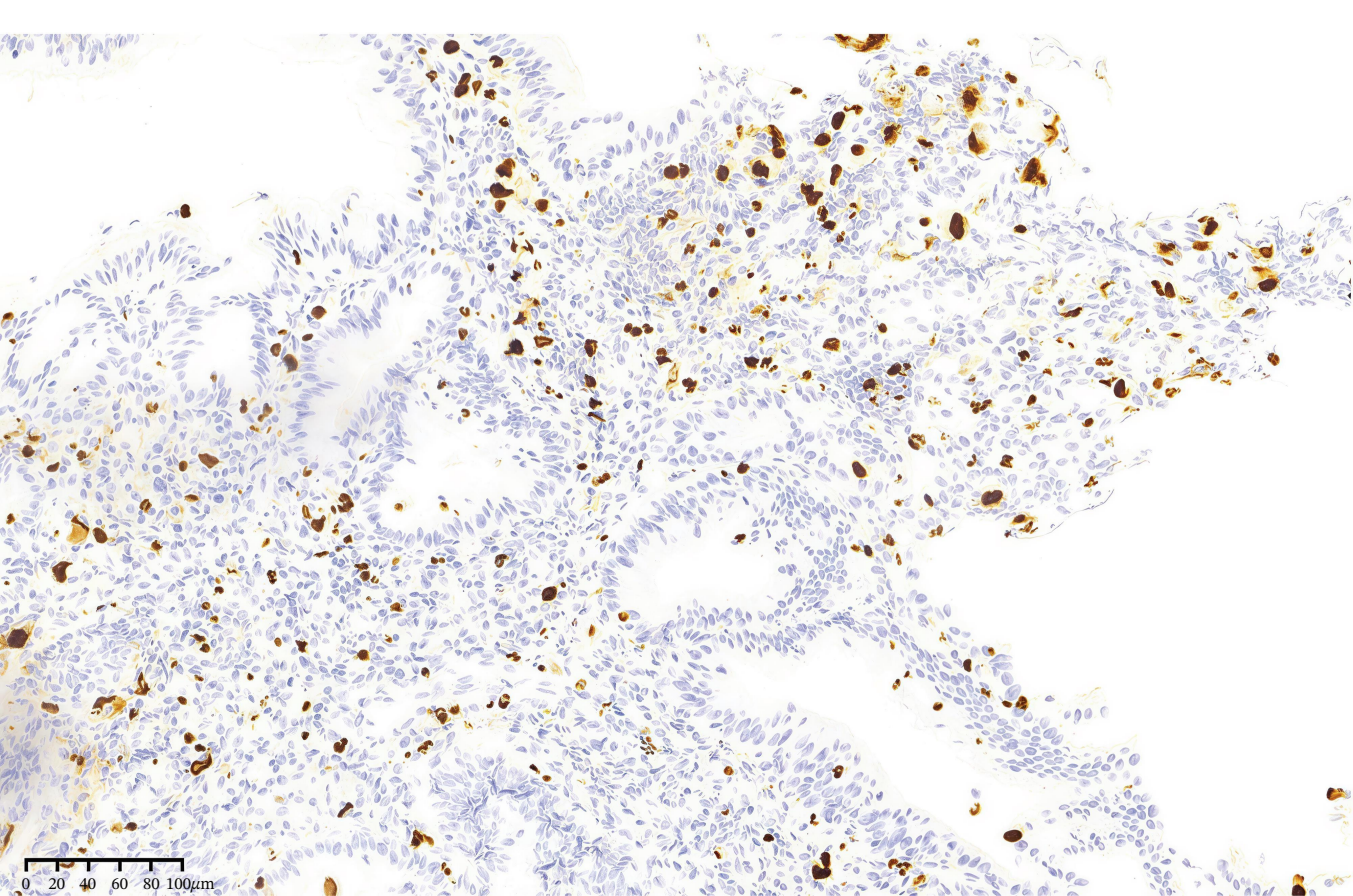
Cytomegalovirus immunostaining was positive, appearing more prominent compared to hematoxylin and eosin staining.

The diagnosis of CMV gastritis, just like intestinal CMV infection, depends principally on laboratory testing due to the variable and nonspecific clinical‐endoscopic presentation. Diagnostic modalities comprised viral culture, CMV‐specific antibody detection, pp65 antigenemia assay, nucleic acid testing (NAT), and histopathological examination [41]. Among these modalities, histopathological examination is generally regarded as the diagnostic gold standard for CMV gastritis. Histopathological hallmarks of CMV‐infected gastrointestinal mucosa include characteristic “cytomegalic” changes, featuring (Figure 1) marked cellular and nuclear enlargement, eosinophilic granular cytoplasm, and intranuclear viral inclusions exhibiting pleomorphism (round, fusiform to irregular morphologies). Some inclusions appear small and are surrounded by a clear halo, forming the characteristic “owl′s eye” appearance [42]. However, in cases demonstrating atypical viral inclusions, IHC staining with CMV‐specific antibodies can be employed. Previous studies have documented that immunostaining effectively highlights CMV‐infected cells, thereby reducing false‐negative results associated with conventional hematoxylin–eosin (HE) staining [43] (Figure 2).

Although CMV gastritis is typically self‐limited, antiviral therapy should be considered for immunocompromised hosts or those with severe/protracted disease in order to improve clinical outcomes [44]. The current first‐line induction regimen consists of intravenous ganciclovir (5 mg/kg every 12 h) for 14–21 days or until documented virologic clearance or significant clinical improvement is achieved [45]. While our systematic review demonstrated clinical improvement in the majority of treated patients, existing literature documents high mortality rates in CMV gastritis (3 months: 20.4%, overall: 40.7%) across both immunocompetent and immunocompromised populations [46]. The observed outcome differences may be attributable to more cases with timely therapeutic intervention in our cohort and incomplete mortality follow‐up in the present study.

CMV‐induced gastrointestinal infection is typically observed in immunocompromised patients and represents a significant cause of mortality in this immunosuppressed population [47]. Therefore, in patients with a history of immunosuppression presenting with gastrointestinal symptoms such as abdominal pain, nausea, vomiting, or gastrointestinal bleeding, prompt endoscopic evaluation is warranted. The possibility of CMV infection should be considered during this assessment, and IHC staining should be performed when indicated to aid in diagnosis.

## Author Contributions

Writing—original draft preparation, Yang Diao; writing—review and editing, Ye Zong, Xun Yang, and Haiying Zhao; HE staining and immunohistochemical staining, Rui Xu and Guangyong Chen.

## Funding

No funding was received for this manuscript.

## Disclosure

All authors have read and agreed to the published version of the manuscript.

## Ethics Statement

The authors have nothing to report.

## Consent

The authors have nothing to report.

## Conflicts of Interest

The authors declare no conflicts of interest.

## Data Availability

Data sharing is not applicable to this article as no datasets were generated or analyzed during the current study.
